# Geriatric nutritional risk index as a prognostic marker for patients with advanced or metastatic urothelial carcinoma receiving immune checkpoint inhibitors

**DOI:** 10.3389/fnut.2026.1857153

**Published:** 2026-06-29

**Authors:** Li-Wen Chang, Chia-Yen Lin, Cheng-Che Chen, Chuan-Shu Chen, Jian-Ri Li, Shian-Shiang Wang, Cheng-Kuang Yang, Sheng-Chun Hung

**Affiliations:** 1 Institute of Medicine, Chung Shan Medical University, Taichung, Taiwan; 2 Department of Urology, Taichung Veterans General Hospital, Taichung, Taiwan; 3 Department of Post-Baccalaureate Medicine, College of Medicine, National Chung Hsing University, Taichung, Taiwan; 4 School of Medicine, National Yang Ming Chiao Tung University, Hsinchu, Taiwan; 5 Department of Medicine and Nursing, Hungkuang University, Taichung, Taiwan; 6 Department of Applied Chemistry, National Chi Nan University, Nantou, Taiwan

**Keywords:** geriatric nutritional risk index, immune checkpoint inhibitor, overall survival, systemic therapy, urothelial cell carcinoma

## Abstract

**Purpose:**

Predicting the clinical outcomes of patients with advanced or metastatic urothelial carcinoma (mUC) receiving immune checkpoint inhibitors (ICIs) remains a challenge. This study evaluates the prognostic value of the Geriatric Nutritional Risk Index (GNRI), a simple screening tool based on serum albumin and body weight, in this patient population.

**Methods:**

We retrospectively analyzed patients with mUC treated with ICIs between January 2018 and December 2022. The GNRI was calculated at the treatment baseline. Patients were categorized into malnutritional status (GNRI<92) and normal nutritional status (GNRI≥92). The primary endpoints were overall survival (OS) and progression-free survival (PFS).

**Results:**

67 patients were categorized as normal nutritional status and 18 patients as malnutritional status. The median OS in the normal nutritional status group is 30.0 months compared to 6.5 months in malnutritional status group (*p* < 0.001). Similarly, the median PFS in the normal nutritional status group is 18.1 months compared to 3.4 months in malnutritional status group (*p* < 0.001). Using the multivariate analysis, normal nutritional status was found as a reduced risk of disease progression (Hazard Ratio [HR] = 0.39, 95% Confidence Interval [CI] 0.16-0.92) and death (HR = 0.26, 95% CI 0.1-0.67).

**Conclusion:**

The findings of this study suggest that malnutritional status, as indicated by a low GNRI, is independently associated with both PFS and OS in patients with mUC undergoing treatment with ICI. However, considering the retrospective, single-center design and limited sample size, further validation in larger, multicenter, prospective cohorts is essential to firmly establish the clinical utility in this patient population.

## 1 Introduction

Advanced or metastatic urothelial carcinoma (mUC) has historically been associated with a poor prognosis, with platinum-based chemotherapy serving as the long-standing first-line standard of care (1–3). However, the therapeutic landscape has been revolutionized by the advencemnet of immune checkpoint inhibitors (ICIs), such as Pembrolizumab, Nivolumab and Atezolizumab. Clinical trials, including KEYNOTE-045, CheckMate 275 and IMvigor211, have demonstrated that ICIs significantly extend overall survival (OS) and improve durable response rates in both platinum-pretreated and cisplatin-ineligible patients (4–6). Combination therapy with pembrolizumab plus enfortumab vedotin or gemcitabine–cisplatin plus nivolumab have been proved to be standard of care for mUC (7, 8). Despite these breakthroughs, clinical response to ICIs remains heterogeneous, necessitating the identification of reliable biomarkers to optimize patient selection and predict treatment outcomes.

In recent years, the intersection of nutritional status and systemic inflammation has gained recognition as a critical determinant of cancer prognosis (9). The Geriatric Nutritional Risk Index (GNRI), originally developed by Bouillanne et al. in 2005, is a simplified objective screening tool derived from serum albumin levels and the ratio of actual to ideal body weight 10. Unlike traditional nutritional assessments, the GNRI is particularly suited for elderly populations and oncological settings due to its ease of calculation and high reproducibility (10). The GNRI has demonstrated broad clinical utility in predicting both perioperative complications and long-term oncological outcomes for patients with solid tumor receiving definitive radical resection (11–15). It has also been adapted to metastatic cancer receiving systemic chemotherapy or ICIs (16). Collectively, these findings highlight the GNRI as a robust, easily accessible predictor of clinical outcomes across various oncological contexts, including advanced disease treated with ICIs.

While its prognostic value has been validated in various solid tumors, its specific role in predicting the efficacy and survival of mUC patients undergoing ICI therapy warrants further investigation. This study aims to evaluate whether baseline GNRI can serve as a robust predictor of OS and progression-free survival (PFS) in this clinical context.

## 2 Patients and methods

### 2.1 Patient selection

The present study was conducted as a retrospective chart review and received formal approval from the Institutional Review Board of Taichung Veterans General Hospital (IRB No. CE13240A-3), with written informed consent obtained from all participants. All research protocols adhered to the relevant clinical guidelines and institutional regulations. Malignancy staging was defined based on the updated American Joint Committee on Cancer (AJCC) and International Union for Cancer Control (UICC) TNM classification system (17). Histopathological grading was performed according to the 1973 World Health Organization (WHO)/International Society of Urologic Pathology (ISUP) consensus criteria (18).

### 2.2 Surveillance protocol

All patients had histologically confirmed advanced or metastatic urothelial carcinoma, with disease staging determined via computed tomography (CT). Participants received first-line immune checkpoint inhibitors (ICIs)—including pembrolizumab, atezolizumab, nivolumab, or durvalumab—administered either as monotherapy or in combination with cisplatin-based chemotherapy. The specific therapeutic regimen was determined by the treating physician’s discretion, taking into account the patient’s renal function.

### 2.3 The geriatric nutritional risk index

The GNRI was calculated as formula: GNRI = 1.489 × serum albumin level (g/L) + 41.7 × (actual body weight [kg]/ideal body weight [kg]) (19). The ideal body weight was identified as [height (m)]^2^ × 22 (kg/m^2^). The value of the actual body weight/ideal body weight was set to 1 when the actual body weight exceeded the ideal body weight. Consistent with established literature, a GNRI threshold of 92.0 was utilized to define nutritional status. Accordingly, patients were categorized as having either normal nutritional status (GNRI ≥ 92.0) or malnourishment (GNRI < 92.0) (15, 20).

### 2.4 Patient characteristics

Patient characteristics included the following: age at ICIs, gender, Eastern Cooperative Oncology Group (ECOG) performance status, Body Mass Index (BMI, kg/m^2^), primary tumor site, metastatic site, smoking status, hemoglobin, platelet, lactate dehydrogenase (LDH), albumin, ICIs treatment protocol and ICIs cycles.

### 2.5 Outcome assessment and statistical analysis

Study endpoints included progression-free survival (PFS) and overall survival (OS). PFS was defined as the interval from treatment initiation to the first documentation of disease progression, according to the Response Evaluation Criteria in Solid Tumors (RECIST) version 1.1, or death from any cause. OS was defined as the time from the start of treatment until death from any cause. For Baseline characteristics, continuous variables were compared using the Mann–Whitney U test, while categorical variables were analyzed using Pearson’s chi-squared test. Survival outcomes were estimated using the Kaplan–Meier method and compared via the log-rank test. To identify independent prognostic factors, univariate and multivariate Cox proportional hazards regression models were employed to calculate hazard ratios (HRs) and 95% confidence intervals (CIs). Due to the limited number of cases and clinical events within individual treatment arms, different immunotherapy regimens were consolidated into two simplified categories (“IO monotherapy” and “IO combination therapy”) to maintain statistical power and prevent model overfitting. Variance Inflation Factors (VIF) examination was also used in the multivariable model to assess collinearity of each variable. Statistical analyses were performed using IBM SPSS Statistics software, version 22.0 (IBM Corp., Armonk, NY, USA).

## 3 Results

### 3.1 Patient and characteristics

A total of 85 patients were enrolled and stratified into two groups: the malnutritional group (GNRI < 92, *n* = 18) and the normal nutritional group (GNRI ≥ 92, *n* = 67), as summarized in Table 1. The median age was 72.0 years (Interquartile Range, IQR: 62.6–77.6) in the malnutritional group and 67.3 years (IQR: 60.0–74.8) in the normal group (*p* = 0.282). Patients in the malnutritional group exhibited a significantly lower median GNRI (89.35 vs. 99.7, *p* < 0.001), poorer ECOG performance status (*p* = 0.009), and lower BMI (*p* = 0.003). No significant differences were observed regarding comorbidities or smoking status. The distribution of primary tumor sites was similar between the malnutritional and normal groups: bladder (44.4% vs. 50.8%), upper urinary tract (50.0% vs. 44.8%), and synchronous involvement (5.6% vs. 4.5%). Metastatic sites included the retroperitoneal lymph nodes, lungs, bone, spleen, adrenals, liver, and mesentery. Notably, bone metastasis was significantly more prevalent in the malnutritional group (27.8% vs. 7.6%, *p* = 0.032). Furthermore, malnourished patients presented with significantly lower hemoglobin (9.85 vs. 11.6 g/dL, *p* = 0.001) and albumin levels (3.3 vs. 4.0 g/dL, *p* < 0.001), alongside an elevated neutrophil-to-lymphocyte ratio (NLR: 8.08 vs. 3.4, *p* < 0.001), higher LDH (290.5 vs. 182.0 U/L, *p* = 0.004), and higher platelet counts (372,000 vs. 263,000/μL, *p* = 0.005).

**Table 1:** Patient characteristics of advanced or metastatic urothelial cell carcinoma receiving first line immune checkpoint inhibitors (*N* = 85).

Characteristics	GNRI < 92 (*n* = 18)	GNRI ≥ 92 (*n* = 67)	*p* value
	*n* (%)	*n* (%)	
Age at ICI (median, IQR)	72.03 (62.56-77.56)	67.30 (59.99-74.76)	0.282
Gender			
Male	10 (55.56%)	44 (65.67%)	0.429
Female	8 (44.44%)	22 (34.33%)	
Performance (ECOG)			0.009**
0	3 (16.67%)	32 (47.76%)	
1	9 (50.00%)	29 (43.28%)	
2	6 (33.33%)	6 (8.96%)	
BMI (median, IQR)	22.32 (19.88-23.85)	24.47 (22.19-26.61)	0.003**
GNRI (median, IQR)	89.35 (83.53-90.84)	99.77 (95.3-104.24)	<0.001**
Primary tumor site^f^			0.909
Urinary bladder	8 (44.44%)	34 (50.75%)	
Kidney and ureter	9 (50.00%)	30 (44.78%)	
Bladder, kidney and ureter	1 (5.56%)	3 (4.48%)	
Distal metastases	11 (61.11%)	28 (41.79%)	0.144
Lymph node metastases^f^	14 (77.78%)	48 (71.64%)	0.768
Lung metastases^f^	6 (33.33%)	17 (25.37%)	0.555
Bone metastases^f^	5 (27.78%)	5 (7.46%)	0.032*
Spleen metastases^f^	0 (0%)	1 (1.49%)	1.000
Adrenal metastases^f^	1 (5.56%)	1 (1.49%)	0.381
Liver metastases^f^	4 (22.22%)	5 (7.46%)	0.090
Mesentary metastases^f^	0 (0%)	2 (2.99%)	1.000
Prostate metastases^f^	0 (0%)	1 (1.49%)	1.000
Smoking^f^	4 (22.22%)	14 (20.90%)	1.000
DM^f^	4 (22.22%)	15 (22.39%)	1.000
Hemoglobin (median, IQR)	9.85 (8-11.23)	11.60 (10.7-13.6)	0.001**
N/L ratio (median, IQR)	8.08 (4.24-14.27)	3.40 (2.08-5.06)	<0.001**
Platelet (*1000) (median, IQR)	372.00 (261.5-463.25)	263.00 (206-322)	0.005**
LDH (median, IQR)	290.50 (202.75-370.75)	182.00 (167-221.25)	0.004**
Albumin (median, IQR)	3.30 (3.18-3.4)	4.00 (3.6-4.2)	<0.001**
ICI therapy			0.231
ICI monotherapy	4 (22.22%)	25 (37.31%)	
ICI combination therapy	14 (77.78%)	42 (62.69%)	
Combination Gemcitabine and cisplatin	8 (44.44%)	37 (55.22%)	0.416
Combination Gemcitabine and carboplatin	5 (27.78%)	4 (5.97%)	0.018*
Combination methotrexate, etoposide and cisplatin	1 (5.56%)	1 (1.49%)	0.381
ICI cycle (median, IQR)	4.00 (3-9)	6.00 (4-13)	0.068
Follow up time (month) (median, IQR)	6.73 (3.88-11.56)	26.32 (11.46-36.79)	<0.001**

Mann–Whitney U test. Chi-Square test. ^f^Fisher’s exact test. *p < 0.05, **p < 0.01.

GNRI, Geriatric Nutritional Risk Index; ICI, immune checkpoint inhibitor; IQR, inter quartile range; ECOG, Eastern Cooperative Oncology Group; BMI, body mass index; DM, diabetes mellitus; N/L ratio, neutrophil/lymphocyte ratio; LDH, lactate dehydrogenase.

Regarding the treatment regimens, there was no significant difference in the distribution of ICI therapy types between the two groups (*p* = 0.231). In the malnutritional group, 22.22% (*n* = 4) received ICI monotherapy and 77.78% (*n* = 14) received ICI combination therapy. Similarly, in the normal nutritional group, 37.31% (*n* = 25) received monotherapy and 62.69% (*n* = 42) received combination therapy.

Among patients receiving combination regimens, the distribution of specific chemotherapy backbones was generally comparable, with the exception of the gemcitabine and carboplatin combination, which was significantly more frequent in the malnourished group (27.78% vs. 5.97%, *p* = 0.018). No significant disparities were observed for the combinations of gemcitabine and cisplatin (44.44% vs. 55.22%, *p* = 0.416) or the triple regimen of methotrexate, etoposide, and cisplatin (5.56% vs. 1.49%, *p* = 0.381).

Furthermore, patients in the malnutritional group completed a lower number of ICI cycles compared to the normal nutritional group, with a median of 4.00 (IQR: 3–9) versus 6.00 (IQR: 4–13), respectively, although this trend did not reach statistical significance (*p* = 0.068).

For ICI regimens in patient population, 47 patients receive atezolizumab, 19 patients receive pembrolizumab, 2 patients receive nivolumab and 17 patients receive durvalumab.

### 3.2 Progression free survival

**Figure 1:**
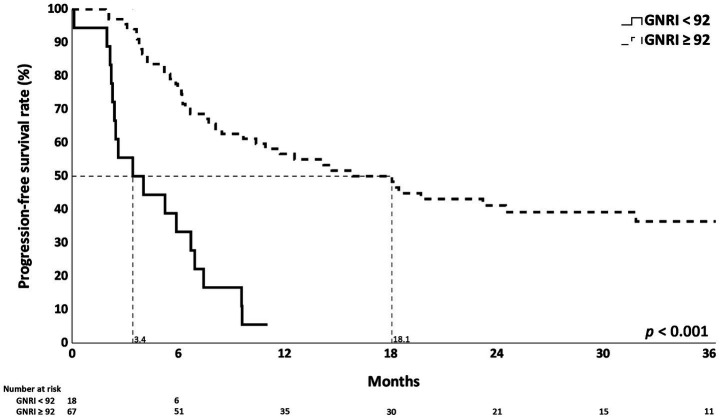
Kaplan–Meier curves of PFS stratified by the GNRI. In mUC patients, those with normal nutritional status (GNRI ≥ 92) achieved a median PFS of 18.1 months, whereas those with malnutrition (GNRI < 92) had a median PFS of 3.4 months (log-rank *p* < 0.001).

Kaplan–Meier analysis (Figure 1) revealed a significant disparity in PFS between the two cohorts; the malnutritional group exhibited a substantially shorter median PFS of 3.4 months compared to 18.1 months in the normal nutritional group (*p* < 0.001).

The results of the univariate and multivariate Cox proportional hazards regression analyses for PFS are presented in Table 2.

**Table 2:** Uni-multivariant for progression free survival.

Variables	Univariate		Multivariate	
	HR (95% CI)	*p* value	HR (95% CI)	*p* value
Age at ICI	1.02 (0.99–1.05)	0.212		
Gender-Male	0.96 (0.56–1.64)	0.891		
Perfomance (ECOG)	2.19 (1.50–3.20)	<0.001**	1.80 (1.19–2.71)	0.005**
Distal metastases	1.56 (0.93–2.63)	0.092		
Lymph node metastases	1.03 (0.58–1.84)	0.913		
Smoking	1.41 (0.75–2.63)	0.283		
DM	0.72 (0.38–1.36)	0.306		
Hb	0.87 (0.77–0.98)	0.023*	1.01 (0.88–1.17)	0.868
N/L ratio	1.10 (1.04–1.16)	<0.001**	1.03 (0.96–1.10)	0.388
Platelet (*1000)	1.00 (1.00–1.00)	0.171		
LDH	1.00 (1.00–1.00)	0.340		
ICI therapy				
ICI monotherapy	1.00			
ICI combination therapy	0.78 (0.46–1.34)	0.370		
GNRI ≥ 92	0.21 (0.11–0.39)	<0.001**	0.27 (0.14–0.54)	<0.001**

Cox regression. *p < 0.05, **p < 0.01.

HR, Hazard ratio; CI, confidence interval; GNRI, Geriatric Nutritional Risk Index; ICI, immune checkpoint inhibitor; ECOG, Eastern Cooperative Oncology Group; BMI, body mass index; DM, diabetes mellitus; N/L ratio, neutrophil/lymphocyte ratio; LDH, lactate dehydrogenase.

In the univariate analysis, several factors were significantly associated with an increased risk of disease progression, including poorer ECOG performance status (HR 2.19, *p* < 0.001), lower hemoglobin levels (HR 0.87, *p* = 0.023) and higher neutrophil-to-lymphocyte ratio (HR 1.10, *p* < 0.001). Notably, a normal nutritional status (GNRI ≥ 92) was strongly associated with a reduced risk of progression (HR 0.21, 95% CI: 0.11–0.39; *p* < 0.001).

After adjusting for potential confounders in the multivariate analysis, two factors remained significantly associated with PFS. GNRI ≥ 92 was identified as a significant independent protective factor against disease progression (HR 0.27, 95% CI: 0.14–0.54; *p* < 0.001). Additionally, ECOG performance status (HR 1.80, 95% CI: 1.19–2.71; *p* = 0.005) were also confirmed as independent prognostic markers. Other variables, including Hemoglobin and neutrophil-to-lymphocyte ratio did not demonstrate significant associations with PFS in this study. Multicollinearity was assessed using VIF for the four variables included in the multivariable model, yielding VIF values of 1.28 for Performance, 1.43 for Hb, 1.57 for N/L ratio, and 1.32 for GNRI ≥ 92, indicating no significant collinearity.

### 3.3 Overall survival

**Figure 2:**
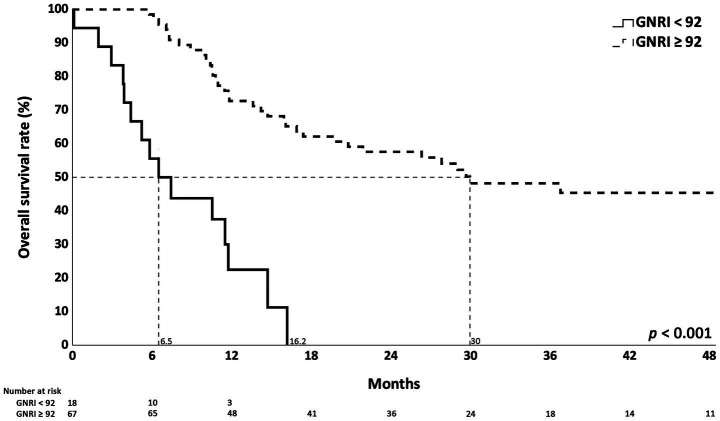
Kaplan–Meier curves of OS stratified by the GNRI. In mUC patients, those with normal nutritional status (GNRI ≥ 92) achieved a median OS of 30.0 months, whereas those with malnutrition (GNRI < 92) had a median OS of 6.5 months (log-rank *p* < 0.001).

Kaplan–Meier analysis (Figure 2) revealed a significant disparity in OS between the two cohorts; the malnutritional group exhibited a substantially shorter median OS of 6.5 months compared to 30.0 months in the normal nutritional group (*p* < 0.001).

The prognostic factors for OS were evaluated using univariate and multivariate Cox proportional hazards regression models, as detailed in Table 3.

**Table 3:** Uni-multivariant for overall survival.

Variables	Univariate		Multivariate		
	HR (95% CI)	*p* value	HR	(95% CI)	*p* value
Age at ICI	1.02 (0.99–1.05)	0.168			
Gender-Male	1.18 (0.65–2.12)	0.585			
Performance (ECOG)	2.37 (1.59–3.52)	<0.001**	1.88 (1.19–2.98)		0.007**
Distal metastases	1.82 (1.04–3.20)	0.037*	1.63 (0.87–3.07)		0.127
Lymph node metastases	1.12 (0.59–2.11)	0.735			
Smoking	1.05 (0.52–2.10)	0.895			
DM	0.65 (0.31–1.34)	0.243			
Hb	0.82 (0.72–0.94)	0.005**	1.02 (0.86–1.21)		0.807
N/L ratio	1.15 (1.08–1.21)	<0.001**	1.06 (0.97–1.15)		0.186
Platelet (*1000)	1.00 (1.00–1.00)	0.178			
LDH	1.00 (1.00–1.00)	0.255			
ICI therapy					
ICI monotherapy	1.00				
ICI combination therapy	0.99 (0.55–1.81)	0.987			
GNRI ≥ 92	0.17 (0.09–0.34)	<0.001**	0.22 (0.10–0.47)		<0.001**

Cox regression. *p < 0.05, **p < 0.01.

HR, Hazard ratio; CI, confidence interval; GNRI, Geriatric Nutritional Risk Index; ICI, immune checkpoint inhibitor; ECOG, Eastern Cooperative Oncology Group; BMI, body mass index; DM, diabetes mellitus; N/L ratio, neutrophil/lymphocyte ratio; LDH, lactate dehydrogenase.

In the univariate analysis, several clinical and laboratory parameters were significantly associated with OS. Poor ECOG performance status (HR 2.37, *p* < 0.001), distal metastasis (HR 1.82, *p* = 0.037), lower hemoglobin levels (HR 0.82, *p* = 0.005) and higher neutrophil-to-lymphocyte ratio (HR 1.15, *p* < 0.001) were all linked to an increased risk of mortality. Conversely, a normal nutritional status (GNRI ≥ 92) was identified as a strong protective factor for OS (HR 0.17; 95% CI: 0.09–0.34; *p* < 0.001).

In the multivariate analysis, after adjusting for variables significant in the univariate model, two factors remained independent predictors of mortality. GNRI ≥ 92 maintained its strong independent prognostic value, significantly reducing the risk of death by 78% (HR 0.22; 95% CI: 0.10–0.47; *p* < 0.001). Other independent risk factors included ECOG performance status (HR 1.88, *p* = 0.007). Distal metastases, hemoglobin and neutrophil-to-lymphocyte ratio did not show independent associations with OS in the final model. Multicollinearity was assessed using VIF for the five variables included in the multivariable model, yielding VIF values of 1.29 for Performance, 1.08 for Distal metastases, 1.43 for Hb, 1.64 for N/L ratio, and 1.33 for GNRI ≥ 92, indicating no significant collinearity.

### 3.4 Subsequent therapy

Table 4 summarizes the distribution of subsequent therapies administered to patients, stratified by their baseline GNRI. 7 of 18 (38.89%) patients in the low GNRI group and 21 of 67 (31.34%) patients in the high GNRI group did not receive subsequent therapy (*p* = 0.545). The most frequently administered subsequent therapy in both cohorts was the Gemcitabine and Cisplatin combination, accounting for 71.43% (*n* = 5) of the low GNRI group and 52.38% (*n* = 11) of the high GNRI group. Other subsequent treatments distributed across the groups included immune checkpoint inhibitors (Atezolizumab, Pembrolizumab, and Nivolumab), Enfortumab vedotin, and Paclitaxel. Overall, there was no statistically significant difference in the choice or distribution of subsequent therapies between the two nutritional risk groups (*p* = 1.000).

**Table 4:** Subsequent therapies administered to patients.

Characteristics	GNRI < 92 (*n* = 18)	GNRI ≥ 92 (*n* = 67)	*p* value
	*n* (%)	*n* (%)	
No Subsequent therapy			0.545
No	7 (38.89%)	21 (31.34%)	
Yes	11 (61.11%)	46 (68.66%)	
Subsequent therapy			1.000
Gemcitabine+Cisplatin	5 (27.78%)	11 (16.41%)	
Atezolizumab	1 (5.56%)	3 (4.48%)	
Pembrolizumab	1 (5.56%)	2 (2.99%)	
Enfortumab vedotin	0 (0.00%)	2 (2.99%)	
Paclitaxel	0 (0.00%)	2 (2.99%)	
Nivolumab	0 (0.00%)	1 (1.49%)	

Chi-Square test. Fisher–Freeman–Halton Exact test. *p < 0.05, **p < 0.01.

## 4 Discussion

The present study demonstrates that the GNRI is a powerful and independent prognostic marker for patients with advanced or mUC receiving ICIs. Our primary finding reveals a profound survival disparity based on baseline nutritional status; patients in the normal nutritional group (GNRI ≥ 92) achieved a median overall survival (OS) of 30.0 months and a progression-free survival (PFS) of 18.1 months, which were substantially superior to the 6.5 months and 3.4 months observed in the malnutritional group, respectively. Multivariate Cox regression analysis further confirmed that a high GNRI serves as a significant independent protective factor, reducing the risk of mortality by 74%. These results underscore the critical impact of host nutritional status on the efficacy of immunotherapy, suggesting that GNRI can effectively stratify risk and guide clinical expectations in the management of mUC.

Table 5 summarized the literature regarding the GNRI in urothelial carcinoma consists of 10 studies spanning various clinical scenarios (20–29). Four of these studies focus on patients with mUC treated with ICIs, specifically evaluating outcomes for those receiving second-line pembrolizumab or general ICI therapy. Another three studies investigate the prognostic impact of GNRI in patients undergoing systemic chemotherapy, including first-line platinum-based regimens, modified gemcitabine/cisplatin, and avelumab switch maintenance. Additionally, three studies examine the role of GNRI in patients with upper tract urothelial carcinoma (UTUC) who underwent definitive surgical interventions such as radical nephroureterectomy or radical nephrectomy. Finally, one study specifically assesses the utility of the index in pretreated mUC patients receiving Enfortumab vedotin. Overall, these studies consistently highlight the GNRI as a significant predictor of CSS, PFS, and OS.

**Table 5:** Geriatric nutritional risk index in urothelial carcinoma.

Author (Year)	Journal	Population (*n*)	Treatment setting	Key findings (Prognostic value)
Etani et al. (2020) (21)	Oncology	Metastatic urothelial carcinoma (mUC) treated previously with platinum-based chemotherapy (*n* = 52)	Pembrolizumab	The median cancer specific survival (CSS) with second-line pembrolizumab therapy was 3.6 months (95% confidence interval [CI]: 2.5-6.1) and 11.8 months (95% CI: 6.2-NA) in the low-GNRI (GNRI<92) and the high-GNRI group (GNRI ≥92) (*p* < 0.01).
Naiki et al. (2021) (22)	Oncology	mUC untreated (*n* = 68)	modified-short hydration gemcitabine and cisplatin	Median overall survival (OS) was calculated as 8.6 (95% CI: 5.4-21.3) and 34.5 (95% CI: 20.5-NA) months for GNRI<92 group and GNRI ≥92 group (*p* < 0.0001).
Isobe et al. (2022) (20)	International Journal of Clinical Oncology	mUC pretreated (*n* = 198)	Second line Immune check point inhibitor (ICI)	The median OS was 18.9 months (95% CI 13.8–26.0) and 4.6 months (95% CI 3.1–6.1) for high and low GNRI groups (*p* < 0.0001). High GNRI as significant prognostic factors affecting long OS (HR: 1.97, 95% CI 1.12-3.45)
Chang et al. (2023) (23)	Scientific Reports	Upper tract UC (*n* = 488)	Radical nephroureterectomy	The 5-year survival rate of disease free survival (DFS), CSS and OS were 48.6, 80.5 and 80.5% in the normal nutritional group and 28.0, 53.2 and 40% in the malnutritional group. Malnutritional status was found as an independent risk factor for OS (HR = 3.94, 95% CI 2.70-5.74).
Wu et al. (2023) (24)	Journal of Cancer Research and Clinical Oncology	Upper tract UC (*n* = 458)	Radical nephroureterectomy	A significantly poorer prognosis was observed in the low GNRI group (*p* < 0.001), with the median period for CSS in the low GNRI group being 46.9 months in comparison to 54.5 months in the high GNRI group. In addition, the OS for UTUC was significantly longer among those with high GNRI as compared to those with low GNRI (*p* < 0.001)
Sugiyama et al. (2023) (25)	Oncology	mUC untreated (*n* = 118)	First line platinum-based chemotherapy	Median OS from the start of first-line chemotherapy was 30.2 months (95% CI: 20.9-NA) for GNRI sustainable and 12.6 months (95% CI: 9.0-21.2) for unsustainable groups, respectively (*p* < 0.05)
Isobe et al. (2025) (26)	Oncology	mUC (*n* = 72)	First line chemotherapy with Avelumab switch maintenance	GNRI sustainability were revealed as significant prognostic factors for OS being Ave-suitable both in univariate and multivariate analysis
Morikawa et al. (2024) (27)	Cancers (Basel)	mUC pretreated (*n* = 61)	Enfortumab vedotin	OS for GNRI ≥ 82 compared to GNRI < 80 in univariate analysis (HR0.36, 95% CI 0.13-0.98, *p* = 0.04). No adjustment in multivariate.
Zheng et al. (2025) (28)	Scientific Reports	Upper tract urothelial carcinoma (*n* = 219)	Radical nephrectomy	GNRI was identified as a significant risk factor for CSS, with patients exhibiting higher GNRI demonstrating elevated CSS (hazard ratio = 0.58; 95% confidence interval, 0.32-0.92; *p* = 0.037)
Sugiyama et al. (2026) (29)	Anticancer research	mUC pretreated (*n* = 105)	Second line pembrolizumab	Median PFS from the start of pembrolizumab was 6.4 months (95% CI = 3.7-9.0) and 3.4 months (95%CI = 2.0-4.1) for GNRI sustainable and unsustainable groups (*p* < 0.01). The median OS was 24.8 months (95%CI = 18.4-40.1) and 7.6 months (95%CI = 3.8-12.6) for sustainable and unsustainable group (*p* < 0.0001).

The therapeutic landscape for mUC has undergone a dramatic evolution over the past decade. Historically, platinum-based chemotherapy—primarily cisplatin-based regimens—served as the exclusive first-line standard of care (1, 30). However, many patients were ineligible for cisplatin due to renal impairment or poor performance status, leaving them with limited options and a poor prognosis (2). The advent of ICIs, such as pembrolizumab, nivolumab, and atezolizumab, has revolutionized treatment by significantly extending OS and providing durable responses even in platinum-pretreated or cisplatin-ineligible populations (4–6). Recent evidence has further solidified the role of combination therapies, such as pembrolizumab plus enfortumab vedotin, as the new standard of care (7). Despite these advancements, the heterogeneous response rates to ICIs remain a challenge, highlighting the urgent need for reliable, easily accessible biomarkers like the GNRI to identify patients most likely to benefit from these modern therapies.

The GNRI was originally introduced in 2005 as a simplified objective tool for assessing nutritional risk in elderly populations, derived from serum albumin and body weight. Unlike traditional subjective assessments, the GNRI offers high reproducibility and ease of use in busy clinical settings (31). Malnutrition represents a prevalent and significant challenge in oncology, as cancer-associated nutritional deficiencies are closely linked to increased morbidity and mortality rates (32). Furthermore, the pathogenesis and progression of malignancies are intrinsically associated with the aging process (33). In addition to oncological characteristics, the patient’s performance status and nutritional reserves significantly influence the selection of appropriate surgical or therapeutic strategies. Consequently, elderly patients are particularly vulnerable to adverse outcomes due to their heightened susceptibility to nutritional impairment.

A low GNRI has been consistently reported as a significant predictor of poor prognosis across various human malignancies, specifically correlating with diminished OS and PFS (10). It has also been extensively linked to postoperative complications across a diverse spectrum of malignancies, including esophageal, gastric, hepatobiliary and colorectal cancers following abdominal surgery (34). Furthermore, its utility extends to orthopedic oncology, where preoperative GNRI has been identified as a significant risk factor for surgical site infections following soft-tissue sarcoma resection (35). Collectively, these findings underscore the GNRI as a robust indicator of nutritional status that is consistently associated with adverse clinical outcomes across multiple human malignancies.

The biological rationale for the association between nutritional status and the efficacy of ICIs lies in the complex interplay between the host’s metabolic health and systemic immunity (36). Malnutrition is often characterized by hypoproteinemia and a depleted state of essential amino acids, which are critical for the proliferation and activation of T-lymphocytes (37). Because ICIs rely on the reinvigoration of the host’s own immune system to target cancer cells, where in malnutritional status, T-cells lack the necessary metabolic reserves to sustain an effective anti-tumor response (38). Furthermore, nutritional impairment is frequently associated with heightened systemic inflammation—evidenced in our study by the elevated neutrophil-to-lymphocyte ratio (NLR) and LDH levels in the malnourished group—which contributes to an immunosuppressive tumor microenvironment (39, 40). This chronic inflammatory state can further exhaust T-cells and diminish the clinical benefit of ICIs, explaining the significantly shorter PFS and OS observed in our malnourished cohort (41).

The clinical utility of the GNRI in urothelial carcinoma has been extensively validated across various therapeutic landscapes, including surgical interventions, systemic chemotherapy, and immunotherapy and summarized in Table 5. The clinical utility of the GNRI has been validated in three separate studies involving patients with upper tract urothelial carcinoma undergoing radical nephroureterectomy. These reports consistently demonstrate that malnutrition, as indicated by a low GNRI, is a significant predictor of poor prognosis, encompassing increased postoperative complications as well as diminished DFS and OS (23, 24, 28). Two literatures reported in patients with mUC treated with first line cisplatin-base chemotherapy, low GNRI patients often experience inferior outcomes. The presence of hypoproteinemia in these individuals serves as a surrogate for low physiological resilience, which ultimately limits the ability to tolerate the rigorous toxicity of chemotherapy (22, 25).

The prognostic utility of the GNRI has been validated in three separate cohort studies involving patients with mUC treated with second-line ICIs, primarily pembrolizumab. The GNRI serves as a robust surrogate for the host’s immune-nutritional status, particularly in elderly populations who often present with diminished organ function and multiple comorbidities. Given that these patients require significant physiological resilience to tolerate and respond to systemic therapy, multivariate analyses have consistently confirmed that a high GNRI—often in conjunction with a low neutrophil-to-lymphocyte ratio—is an independent predictor of prolonged OS (20, 21, 29). These findings suggest that maintaining an optimal nutritional state is essential for supporting the metabolic demands of the immune system during checkpoint inhibition. Similar findings were observed in a cohort of patients receiving avelumab switch maintenance following first-line chemotherapy, where a higher or sustainable GNRI was significantly associated with superior therapeutic response and prolonged overall survival OS (26).

Aligning with the current literature, our principal finding identifies the baseline GNRI as a robust and independent prognostic factor for patients with mUC receiving first-line ICIs. While the therapeutic landscape has shifted toward immunotherapy, our data underscore that host-related factors—specifically nutritional status—remain critical determinants of clinical success. These results suggest that the GNRI can effectively complement traditional clinical parameters, such as ECOG performance status, to provide a more comprehensive risk stratification for patients starting systemic therapy.

Despite the significant findings regarding the prognostic value of the GNRI, this study has several limitations that should be acknowledged. First, this study was conducted as a retrospective chart review at a single institution, which inherently introduces the potential for selection bias and may limit the generalizability of the results to broader populations. Second, the total cohort consisted of 85 patients, with a relatively small number of individuals categorized into the malnutritional group (*n* = 18). This small sample size may reduce the statistical power to detect smaller differences in secondary clinical variables. Third, participants received a variety of ICI regimens (pembrolizumab, atezolizumab, nivolumab, or durvalumab) and different treatment protocols (monotherapy vs. combination therapy). This therapeutic diversity may introduce confounding variables regarding individual drug efficacy. Fourth, the GNRI was calculated only at the treatment baseline. Because nutritional status is dynamic and can change during the course of immunotherapy or chemotherapy, this study did not account for the prognostic impact of GNRI changes (sustainability) over time, which has been highlighted as significant in other literature. Fifth, while the GNRI is a validated objective tool, it does not directly measure muscle mass (sarcopenia) or physical strength (frailty). Incorporating more advanced body composition analysis, such as CT-based skeletal muscle index, might provide a more comprehensive view of the patient’s physiological state. Despite these limitations, our findings underscore the GNRI as a highly convenient and robust prognostic biomarker for survival outcomes in the management of patients with mUC.

## 5 Conclusion

The findings of this study suggest that malnutritional status, as indicated by a low GNRI, is independently associated with both PFS and OS in patients with mUC undergoing treatment with ICI. However, considering the retrospective, single-center design and limited sample size, Further validation in larger, multicenter, prospective cohorts is essential to firmly establish the clinical utility in this patient population.

## Data Availability

The raw data supporting the conclusions of this article will be made available by the authors upon reasonable request.
